# Possibilities for ranking business schools and considerations concerning the stability of such rankings

**DOI:** 10.1371/journal.pone.0295334

**Published:** 2024-02-15

**Authors:** Sandra Boric, Gerhard Reichmann, Christian Schlögl

**Affiliations:** 1 Department of Journals, Databases, and License Management, University Library Graz, University of Graz, Graz, Austria; 2 Institute of Operations and Information Systems, University of Graz, Graz, Austria; Szechenyi Istvan University: Szechenyi Istvan Egyetem, HUNGARY

## Abstract

In this article, we discuss possibilities for ranking business schools and analyse the stability of research rankings using different ranking methods. One focus is set on a comparison of publication-based rankings with citation-based rankings. Our considerations and discussions are based on a (small) case study for which we have examined all (six) business schools at public universities in Austria. The innovative aspect of our article is the chosen mix of methods and the explicit comparison of the results of a publication analysis with those of a citation analysis. In addition, we have developed a new indicator to check the stability of the obtained ranking results with regard to the individual business schools. The results show that the ranks of the individual business schools are quite stable. Nevertheless, we found some differences between publication-based and citation-based rankings. In both cases, however, the choice of the data source as well as switching from *full* to *adjusted* counting only have little impact on the ranking results. The main contribution of our approach to research in the field of university rankings is that it shows that focusing on a single (overall) indicator should be avoided, as this can easily lead to bias. Instead, different (partial) indicators should be calculated side by side to provide a more complete picture.

## Introduction

Direct measures of scientific research output have generated a whole research field called *bibliometrics* [1]. Bibliometric analyses are used, among other things, for national and international research funding procedures, and as a justification for the allocation of research funds [2]. Publication counts are the most traditional bibliometric indicator. They are based on bibliographic attributes such as *authors*, *publication date*, and *publication type* [3]. Using statistical analyses, they can provide insight into scholarly processes, e.g., the growth or decrease of publication rates, the origin and evolution of disciplines, publication policies, and interdisciplinarity [3, 4]. Besides publications, the analysis of citations which stems back to as early as the 1920s [5] is also of high importance for the illustration of scientific performance. Citation patterns and frequency can be used to assess the impact of authors (individuals or groups) or certain publications, and to measure the quality of the latter [4]. Citations are an attractive subject of study due to them being unobtrusive, nonreactive, and readily available [6]. They can serve as an acknowledgement of some other work in the same research field [7, 8], although the intensity of acknowledgeement varies by discipline [9].

Research performance has garnered a considerable interest in different scientific fields [10] and can particularly be measured via the *number of scientific publications* and their citations of individuals, groups of individuals, institutions, regions, countries, or continents [11]. Bibliometric data can therefore serve as a means of rating not only individual researchers [12] but also whole institutions, such as departments [13], faculties of universities (in the following named as schools) [14–16] or universities [17], and even countries [18]. In this paper, we focus on the evaluation of institutions (schools) but limited to a single subject (i.e., business administration).

The evaluation of the research performance of universities, schools, departments, and professors has already been established for several decades in the U.S.A., Great Britain, Australia, and the Netherlands [19]. The measurement of research performance in the form of rankings is particularly widespread with regard to universities [20–22]. University rankings are highly accepted among many stakeholders and the wider public due to their dissemination in the media [23]. Rauhvargers [24] points out that (global) university rankings are often regarded by policy makers and society at large as tools for university transparency. Accordingly, it could be argued that without rankings, universities would not be transparent.

In addition to the numerous literature on the ranking of entire universities, there are also some articles on the ranking of university business schools. Dichev [25] investigated the quality of business school rankings. However, his study was limited to the U.S.A. and two rankings (i.e., Business Week and U.S. News & World Report) that were popular there, but not very scientific. Siemens et al. [26] also examined the quality of one of those two popular press business school rankings (i.e., U.S. News & World Report) by comparing the ranking results there with research productivity in prestigious business journals. Significant correlations indicate satisfactory quality of the former. Kumar and Kundu [27] evaluated 600 business schools from around the world based on their number of publications over a period of ten years. Due to the restriction to publications in three core international business journals, the total number of articles recorded was only 900. (There is also an update of their study [28].) Bickerstaffe and Ridgers [29] discuss one of the (supposedly) leading global business school rankings (i.e., Economist Intelligence unit’s *Which MBA*?). However, that ranking is based exclusively on MBA programs, and research performance plays no role at all. Bradshaw [30] and Devinney et al. [31] deal with the Financial Times business school rankings, which at least include the faculty’s research performance. Among other things, those 40 journals that are used to determine research performance are listed. These are exclusively business (management) journals. The study by Baden-Fuller et al. [32] was also limited to 32 journals from four business administration areas (finance, human resources, management, and marketing). In their study, all European business schools were ranked based on their research performance in the form of publications in those journals. Since the relevant journals were exclusively English-language journals, it is not surprising that seven of the top ten business schools are from the U.K. The relatively recent article by Lozano et al. [33] has again only little research relevance. It states that the existing well-established rankings practically force individual business schools to create similar curricula and to follow similar strategic approaches in order to be competitive in those rankings.

All these articles on business school ranking, which were mainly published in business (management) journals (e.g.: Management International Review, Journal of Management Development, and Journal of Business Research), are usually not very detailed and often only treat research performance marginally. In our literature analysis, we thus focused on publications on ranking entire universities.

One topic that is certainly of great importance related to the quality of business schools is accreditation. The best-known accreditation organizations include the Association to Advance Collegiate Schools of Business (AASCB) in the U.S.A., the Association of MBAs (AMBA) in the U.K., the European Foundation for Management Development (EFMD) in Europe, and the Foundation for International Business Administration Accreditation (FIBAA) in German-speaking countries. Although the priorities set there for accreditation are quite different, the focus is always on (MBA) study programs. This is particularly evident at the AMBA, as this organization does not accredit entire business schools, but only individual study programs. What all of the above-mentioned accreditations have in common is that the focus is always on the (MBA) study programs, especially on their content and framework conditions. Good research is seen primarily as a framework for a good study program and is therefore only considered marginally. The quality of research is usually not measured in great detail based on publications in renowned journals or on the basis of the citations received. Due to these circumstances, we did not analyse the literature on accreditation of business schools in detail.

Various (potential) problems that have especially been discussed in the context of university rankings can also appear in other institutional rankings in the university sector. This includes in particular the frequent focus on a single ranking method [34] and the disregard of size effects [21]. The former can lead to quite arbitrary ranking results, even if not entire universities but only sub-units such as departments or–as in our case–schools are examined. This problem can be solved by using a mix of methods for ranking. We took this argument into account for our case study by examining different variants for a ranking at school level, which we eventually do not combine into overall rankings, but compare from the point of view of *stability*. The latter (i.e., the disregard of size effects) inevitably leads to a preference of ‘larger’ universities. Since the focus of a research evaluation based on publications and citations will naturally be on the number of researchers, this preference becomes greater the more the compared universities differ in terms of the input factors. This problem can be easily solved by using size-independent rankings, i.e., dividing research performance by the number of associated researchers. We have therefore only carried out size-independent rankings in our case study, with two exceptions that are intended to showcase this problem.

The use of different ranking methods may lead to diverging results. However, we found a particular lack of stability-related discourse in research output analyses and academic institution rankings. Dobrota & Dobrota [35] took the *Shanghai Academic Ranking of World Universities (ARWU)* ranking results based on six university performance indicators and compared them to ARWU ranking results without two of those six indicators, namely the Nobel prize and the fields medal indicators. They compared, i.a., the stability of the two rankings. The comparison showed a greater stability when leaving out the two indicators, with ranks being tightly spread throughout the positions. Dobrota et al. [36] even proposed their own ranking method. Using the *Quacquarelli Symonds World University Rankings (QS)*, they showed that this leads to improved stability. Waltman et al. [34] explored the rankings of the *Centre for Science and Technology Studies at Leiden University (Leiden)* and compared its methodology to other university rankings such as the ARWU and the *Times Higher Education World University Rankings (THE)*. What sets the Leiden Ranking apart is, i.a., the use of stability intervals which show an indicator’s sensitivity to changes in the underlying set of publications [34]. Dobrota & Jeremic [37] compared the *QS* ranking to the *University Ranking by Academic Performance (URAP)* in terms of the stability of their ranking methods. They showed that the latter provides more stable results.

An analysis of previous rankings in the university sector shows that these were often either publication-based [13, 28, 38] or citation-based [39–41]. Many rankings also combine publication- and citation-based data [12, 15–17, 42]. However, we did not find any research that explicitly compares publication-based to citation-based rankings. Such a comparison presupposes that the procedure is as similar as possible in both cases. This means, for example, that the same data source (e.g., Web of Science) is used, that co-authorships are treated in the same way (e.g., full counting), or that in both cases, ranking is either size-dependent or size-independent. The main goal of our study is to close this research gap by comparing indeed comparable versions of publication- and citation-based rankings. This comparison using a specially developed stability index is also intended to provide insights into whether the use of a single (overall) ranking indicator can be sufficient or whether it would be better to use several (partial) indicators side by side.

In Germany, an early conducted bibliometric and content analysis is that of Spiegel-Rösing [43] who showed that research productivity varies widely between institutions [44]. Other early research evaluations conducted in Germany are, i.a., a study by Heiber [45] as well as a study by Pommerehnel and Renggli [19]. We chose Austria for our exemplary analysis due to the lack of bibliometric analyses with a focus solely on Austria conducted so far–most bibliometric analyses include Austria only in combination with Germany and Switzerland [44, 46, 47].

Using data retrieved from WoS and Scopus at *university professor* level which is then aggregated at university (school) level, our goal is to answer the following five research questions:

1. How visible is business administration research at the considered universities in WoS and Scopus?
2. How can universities be ranked based on their business administration research?
3. How stable are the ranking results with regard to the ranks of the individual business schools?
4. How stable are the ranking results in case of ranking method variations, in particular when switching from publication-based to citation-based rankings?
5. Is the use of a single (overall) indicator sufficient for a university ranking?

## Literature review on international university rankings

The following literature overview presents common international university rankings considered for the derivation of several indicators for our empirical study. With regard to the research performance of universities, several international university rankings have been developed so far and have already been analysed and compared in scientific literature. Table 1 contains selected studies and shows which international ranking systems they have used or compared. (A comprehensive review of international university rankings is provided by Rauhvargers [24]).

**Table 1:** Selected studies (arranged chronologically by year of publication), including information about which international ranking system(s) they use or compare.

Reference	ARWU	CHE	Leiden	QS	SIR	THE	UMR	U.S. NWR	Other
Marginson & Wende (2006) [48]	X					X			
Buela-Casal et al. (2007) [22]	X					X			X
Aguillo et al. (2010) [21]	X		X						X
Lukman et al. (2010) [49]	X	X							X
Raan et al. (2011) [39]	X		X	X		X			
Rudinger & Hilger (2011) [50]		X							
Chen & Liao (2012) [51]	X					X			X
Soh (2011) [52], (2013) [53], (2015) [54]	X			X		X			
Waltman et al. (2012) [34]	X		X		X	X			
Paruolo et al. (2013) [55]	X								X
Horstmann & Hachmeister (2016) [56]		X							
Piro & Sivertsen (2016) [57]	X					X			
Cantu-Ortiz (2017) [58]				X	X		X	X	X
Dobrota & Jeremic (2017) [37]				X					X
Moed (2017) [59]	X		X	X		X	X		
Olcay & Bulu (2017) [60]	X		X	X		X			X
Vernon et al. (2018) [20]	X		X	X	X	X	X	X	X
Fauzi et al. (2020) [61]	X		X	X		X			X
Selten et al. (2020) [62]	X			X		X			
Gadd et al. (2021) [63]	X		X	X		X	X	X	

For our analysis, we chose systems that occur the most often in bibliometric respective scientometric literature. Using data collected via secondary sources such as official websites, we analyzed the methodologies of the following important [64] rankings: (Rankings of the) *Centre for Science and Technology Studies at Leiden University (Leiden)* [65], *Quacquarelli Symonds World University Rankings (QS)* [66], *Shanghai Academic Ranking of World Universities (ARWU)* [67], and the *Times Higher Education World University Rankings (THE)* [68]. (The THE and QS rankings were used to jointly publish the *THE-QS World University Rankings* between 2004 and 2009. After the end of this collaboration, QS kept using the methodology of these joint THE-QS rankings, and since 2010 these rankings are known simply as the *QS World University Rankings*. In the meantime, THE together with Thomson Reuters developed a methodology for another ranking called *THE World University Rankings*, and began publishing it in 2010. Both rankings use data from Scopus [59]. Rather than on research data and productivity, the THE-QS World University Rankings depended on a survey’s goodness and representativeness, and were strongly biased [21].) We furthermore included the *CHE Ranking* [69], the *SCImago Insitutions Rankings World Report (SIR)* [70], the *U-Multirank World University Ranking (UMR)* [71], and the *U*.*S*. *News & World Report University Rankings (U*.*S*. *NWR)* [24, 58, 72].

The ARWU and Leiden rankings are strongly based on research data and productivity [21], and in the Leiden, ARWU, THE, and QS rankings, *citations* play a crucial part [39]. Lukman et al. [49] provided an overview of ranking systems and showed that the ARWU, CHE, and THE rankings include *publication*-based indicators in particular, which is why we chose those systems for further analysis. The stated purpose of the ARWU, Leiden, SIR, THE, and UMR rankings is *research performance*, and all of them intend to be used for comparisons between institutions and countries [20].

Many of those ranking systems have also turned towards *subject*-based rankings [37]. We included the CHE ranking in particular since it contains subject-specific comparisons of selected research institutions in Germany, Switzerland, the Netherlands, but also Austria, ranked among others by *business administration* [50, 56, 73]. (Just like Olcay & Bulu [60] and Waltman et al. [34], we did not include the *Webometrics Ranking of World Universities*, a ranking system entirely based on webometric indicators [34] that is commonly found in some of the literature that analyses university ranking systems. Olcay & Bulu [60] argue that about half of the indicators the Webometrics depends on requires visibility to data that are not measured in any other leading indices.)

Table 1 reveals that in the 20 studies examined, the ARWU ranking was used most frequently (16 times), followed by the THE (13 times), QS (10 times) and Leiden (8 times) rankings. The remaining rankings (with the exception of ‘Other’) were only used 3 to 4 times.

Although the studies listed in Table 1 contain comparisons of various ranking systems, a direct comparison of different ranking systems’ indicators proves to be difficult due to the non-systematicity of their used categories, indicators, and methodologies. Definitions and criteria can also vary depending on the ranking system [74]. The circumstances regarding the choice of a specific indicator or evaluation methodology, the validity check, and the parties responsible for criteria decision (and the decision transparency) are often also unclear [49, 74, 75]. Different ranking systems use different indicators, and although this means that they thus automatically produce different ranking results, the overall ranking patterns are not as dispersed since the used indicators are mainly focused on a few general categories such as *finances*, *education*, *student body and graduates*, but also *research excellence and reputation* [76].

As the second part of the literature review, we analysed the occurrence of different ranking indicators in the eight international ranking systems considered in order to find suitable indicators for our study. Even though there are many difficulties in direct comparisons of overall ranking results, there is at least a reoccurrence of *research performance*-measuring indicators in various ranking systems. Publication-based and citation-based indicators used in our study are listed in Tables 2 and 3. We laid them out as equivalents to indicators that appear in various international ranking systems.

**Table 2:** Publication-based ranking indicators used in our study, and equivalents found in international university ranking systems.

Publication-based indicators		
used in our study	found in international ranking systems	
	ranking system name	description
Total number of publications	ARWU	Research Output: Number of research articles published in Nature and Science, and number of articles indexed in WoS (SCIE and SSCI 2022) [34, 52, 53, 57, 60, 67, 77]
	Leiden	P: Total number of publications of a university [65] based on WoS [34]
	SIR	Output: Total number of documents published in scholarly journals indexed in Scopus [70]
	UMR	Research: Research publications (absolute numbers) [78]
	U.S. NWR	Publications: Total number of scholarly papers (reviews, articles and notes) [72]
Number of publications per researcher	CHE	Publications per professor in the *business administration* field [69]
	THE	Research productivity: Number of publications per scholar (published in the academic journals indexed by Scopus) [57, 60, 79]
Relative number of researchers with five or more publications found	QS	A minimum publication threshold is set [66]

**Table 3:** Citation-based ranking indicators used in our study, and equivalents found in international university ranking systems.

Citation-based indicators		
used in our study	found in international ranking systems	
	ranking system name	description
Average citation count–citations per researcher	QS	Citations per faculty member [39, 52, 53, 60]
	THE	Research Influence: Average number of times a university’s published work has been cited in Scopus in 2017–2022 [57, 79]
Average citation count–citations per publication	CHE	Citations per publication [69]
	Leiden	MCS: Average number of citations of the publications of a university [39, 65] based on WoS [34]
	QS	Research citations per paper [37, 39, 66]
	UMR	Research: Citation rate [78]
	U.S. NWR	Normalized citation impact: Total number of citations per paper [72]

## Methodology

As the subject of our research, we have chosen Austrian public universities–more precisely the business schools of these universities. We collected our data based on the relevant (business administration) professors. Aggregated data of individual researchers enable the evaluation of whole institutions, such as schools [80]. Our chosen to-be-analysed institutions are schools of public universities located in Austria that offered a bachelor’s or master’s degree in business administration at the time of data retrieval. We did neither consider private universities, technical universities, nor so-called ‘Fachhochschulen’ (i.e., universities of applied sciences) due to a lack of comparability and to avoid differences in results stemming from differences in institution type. The application of these criteria leads to six business schools in Austria being suitable for our analyses. Table 4 shows these six schools selected for our study, and their associated universities. The Vienna University of Economics and Business (hereinafter abbreviated with VUEB) does not have a school structure, since (almost) all departments are assigned to the business administration area.

**Table 4:** Universities (schools) chosen for this study.

University name	School name	No. of researchers (professors) chosen for analysis
University of Graz	Business, Economics, and Social Sciences [81]	34
University of Innsbruck	Business and Management [82]	41
University of Klagenfurt	Management and Economics [83]	20
University of Linz	Social Sciences, Economics and Business [84]	48
University of Vienna	Business, Economics and Statistics [85]	37
Vienna University of Economics and Business (VUEB)	Vienna University of Economics and Business (VUEB)	103

Regarding the choice of researchers, similar to Fabel et al. [47], we considered all Austrian business administration professors who have a professor’s title (including associate and assistant professors) and hold a permanent position at a university. We also considered professors on leave or maternity leave, provided the leave was only temporary. Also, to prove that one of their main tasks lies in carrying out research, each considered researcher had to have a completed doctorate. Our chosen researchers had to be employed at one of the six universities as of 31^st^ of December 2016. We excluded emeritus professors and honorary professors since at the time of data collection, the assignment of their research performance to a university was not clearly ascertainable. We further excluded lecturers since they usually do not produce research output. Table 4 further lists the number of professors assigned to each school. We analysed a total of 283 researchers.

Research output studies should cover at least three to five years in order to eliminate outliers [44]. Past bibliometric studies have often used a time span of at least ten years [44, 46, 86–89]–this goes for even older studies [90–93]. Based on these previous studies, we have also set the time period for our study to ten years, namely from 2008 to 2017.

As our data sources, we chose the databases *Web of Science* (*WoS*) and *Scopus*, because they are well established and contain both publication and citation data. In general, Scopus is found to have a better coverage than WoS–e.g., when it comes to articles with citations [94], the total scientific and scholarly publication output [95], and the coverage of English as well as non-English publications [96].

In both databases, the data is searchable for a number of search fields. Some of the most crucial search fields in WoS are *topic*, *title*, *author*, *publication name*, *year published*, and *language* [97]. Just like in WoS, the query-settings in Scopus can be used to refine the search by various fields such as *document type*, *author*, *affiliation*, *title*, *published year*, *language*, or even *funding sponsor* [98]. We did not consider *Google Scholar* because WoS and Scopus are primarily designed for capturing citations and have tools to uniquely identify authors [99], whereas Google Scholar does not meet these requirements.

From WoS and Scopus, we retrieved data of each relevant researcher’s articles and reviews with a publication date of 2008 to 2017. Our (manual) data retrieval lasted from January to April 2019. Our focus was set on articles and reviews published in academic journals only [100, 101]. Even though other publication types such as books can be reputation-enhancing for a researcher, the heterogeneous nature of books and publishers turns the derivation of objective quality measures into a difficult task [102]. In contrast, academic journals provide a valid context for reliable quality measurement. Since the 1980s, economics departments have therefore been ranked based on journal articles in almost all studies [103, 104].

In the WoS Core Collection, we limited our search to the Science Citation Index Expanded (SCIE) and the Social Sciences Citation Index (SSCI). We restricted document types to articles and reviews in all languages available. To create an initial search query, we used the first forename and the surname of each professor. Authors’ middle initials and further forenames can occur in various forms and can be treated differently in the literature published by those authors [105], which is why we excluded them since they potentially limit our obtained number of hits. For example, our initial search query for Christian Schlögl from the University of Graz was set as follows:

*AU = (Schlögl Christian OR Schloegl Christian OR Schlogl Christian)*

Similar to, e.g., the Swedish language [106], German-language names can contain diacritics and other special signs as input data, such as *ä*, *ö*, *ü*, and *ß*. To combine possible variations of the same researcher’s name, we therefore used the Boolean operator ‘OR’ in our search queries, as shown above. We also used ‘OR’ for queries of individuals with double surnames (e.g., *AU = (Sommersguter-Reichmann Margit OR Sommersguter Margit OR Reichmann Margit)*) and of individuals who had their surnames changed, e.g., as a result of marriage.

However, since our initial search query did not contain any identifier of authors (such as an author ID), we nevertheless ran the risk of not knowing whether two identical author names denote the same person or two different persons [106]. Thus, we used filters to exclude all research categories but *business administration* and *multidisciplinary sciences* (the latter category might also contain some business administration-related publications). We then manually went through the obtained results list and excluded *multidisciplinary sciences*-entries if they did not fit the business administration research field. We further examined the remaining entries in the results list for their associated institutions listed in the author information of each entry. However, since authors might have published from institutions other than the one, they were currently affiliated with, we did not automatically exclude such entries via the WoS filter *Organizations-Enhanced*. Instead, we used each author’s publication website to double-check the entries. If publications from the results list in WoS were nowhere to be found neither on the respective authors’ homepages, in their institution’s research documentation databases, nor on other external sites (such as their *ResearchGate* or *LinkedIn* profiles), we excluded those entries.

To enable a comparison of the results of both databases, we retrieved data from Scopus in a manner as similar as possible to WoS. We streamlined our search process in Scopus to find each professor’s *Scopus author-ID* (in the results list, a click on the author’s name leads to the author’s page where the author-ID can be found) and then inserting that ID into a specified search query to obtain a hit list. We used both the Scopus field code *Author Name* and *Author* (for further explanation, refer to the sub-section *S1 Scopus field codes* in the S1 Appendix) in each professor’s initial search query to first retrieve their particular Scopus author-ID. We then set our search query string for each professor as follows:

*AU-ID (*[insert 11-digit Scopus author-ID of each professor]*) AND DOCTYPE (ar OR rev) AND (PUBYEAR > 2007 AND PUBYEAR < 2018)*

For each publication, we retrieved its publication year, publication language, page number, number of co-authors (including the researcher), and number of citations (including and excluding self-citations) in WoS and Scopus. We specifically included self-citations to calculate the self-citation rate, since especially for smaller journals, self-citations can serve as the main source of citations [107]. For each author, we further retrieved their *h*-index from WoS and from Scopus.

To answer research question 1, we use the data collected to determine how many of the relevant researchers have at least one publication in WoS or Scopus for the set observation period. The share of these researchers in relation to all relevant researchers from the same school is referred to as visibility of business administration research in the following.

To answer research question 2, we carry out various rankings for our study objects (6 business schools with a total of 283 relevant researchers), which can be found in the literature in the same or a slightly modified form (see below). We differentiate between publication-based and citation-based rankings. Within these two categories, we vary the data source (WoS or Scopus) and the treatment of co-authorship (full or adjusted counting). As part of the publication-based approach, we also carry out some size-dependent rankings and rankings based on the number of pages published. Both are primarily used for demonstration purposes and are not recommended by us for performance comparisons carried out in practice due to various shortcomings that will be discussed in the *Results and Discussion* section. As part of the citation-based approach, we also carry out some rankings that do not use the number of researchers as a measure of size, but the number of publications (written by the relevant researchers and contained in WoS or Scopus). We also present four rankings based on the *h*-index. It also applies to these two additions that we would not recommend such rankings for evaluation practice–we elaborate on this in the *Results and Discussion* section. In that section, we further analyse the main influencing factors on all these rankings.

In our study, we include the following indicators that we found being used in one or more of the eight international ranking systems, as well (see Tables 2 and 3):

- **Total number of publications**
- **Number of publications per researcher**
- **Relative number of researchers with five or more publications**
- **Average number of citations per researcher**
- **Average number of citations per publication**

Furthermore, we include the following indicators which we did not find being used in any of the eight international ranking systems, and we explain our choice:

- **Proportion of researchers with publications found (in % of total)**

Coverage in databases such as Scopus and WoS is often taken into consideration for international ranking systems such as ARWU (WoS) [34, 53, 77], Leiden (WoS) [34], SIR (Scopus) [70], THE and QS (Scopus) [59]. And although bias in these databases has already been pointed out in the literature (see, e.g., [94–96, 99]), the *proportion of researchers with publications found (in % of total)* in these databases is still considered a crucial indicator for the research performance of a university, as there is no practical alternative.

- **Number of pages per researcher**

We use the average number of pages per researcher to see whether there are business schools whose researchers tend to publish longer journal articles. However, it should be noted that the number of pages fluctuates relatively strongly depending on the article format or magazine format, which means that it does not make sense to include the number of pages in stability calculations.

- **Relative number of researchers with ten or more publications found**

The *relative number of researchers with ten or more publications found* is related to the aforementioned *proportion of researchers with five or more publications*, but this time with a different limit. Over the ten-year observation period set for our study, this results in an average of at least one publication per year. We consider the use of an indicator that measures the number of (highly) active researchers to be very relevant for university rankings.

- **Aggregated number of publications of top five researchers & aggregated number of citations of top five most cited researchers**

Focusing a research evaluation on ‘top researchers’ does occur in evaluation practice, but is often only used in *citation* analyses [20]. We also suggest using a corresponding indicator with regard to *publication* analyses. However, in both cases it should be noted that the pool of possible top researchers (we use the top five researchers in our study) at smaller institutions is inherently smaller due to the smaller number of researchers. As part of the analysis of the eight international rankings systems we found similar indicators. In the ARWU ranking, we found the indicator ‘highly cited researchers’ [52, 53, 67, 77], and the UMR ranking and the U.S. NWR ranking take into account the most frequently cited publications [72, 78].

- **Average *h*-index per researcher & *h*-index per business school**

We also include the *h*-index in our university rankings since it combines publication as well as citation data. It can be measured not only at the level of individual researchers (*average h-index per researcher*), but also at the level of institutions such as business schools. However, the latter (*h-index per business school*) is size-dependent and therefore not suitable for stability comparisons.

**Fig 1:**
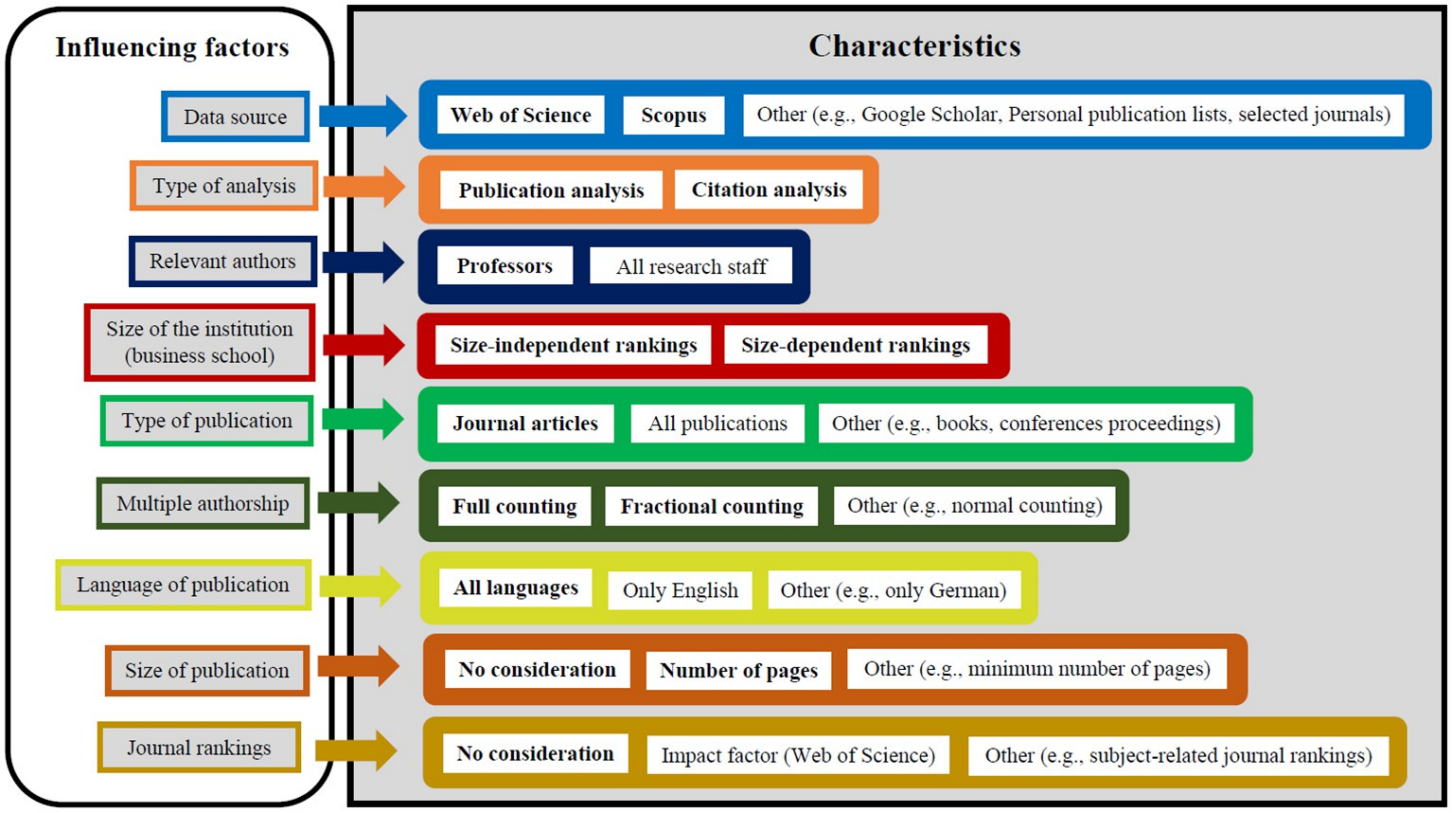
Model of ranking variants for business schools.

Finally, with regard to research question 2, Fig 1 is based on [108] and shows which variations can be made with regard to the indicators just presented and which ones we actually made (see also above). Of the many possible variants (combination of one characteristic of each influencing factor), we have only made variations with regard to five possible influencing factors (all used characteristics are highlighted in bold in Fig 1): data source, type of analysis, size of the institution, multiple authorship, and size of publication. And even with regard to these five factors, we only considered two possible characteristics per factor. However, we did not vary the other four influencing factors: With regard to the relevant authors, only professors were considered, with regard to publication types we only used journal articles, with regard to language of publication we made no restrictions, and we generally did not take journal rankings into account.

In order to check the stability of the ranking results (research questions 3 and 4), we first compare the ranks achieved by each business school based on the different approaches in tabular form. To answer research question 3, we also measure the stability of the rankings with regard to the individual business schools using a stability indicator developed specifically for this study. This is similar to the standard deviation calculation but, in contrast, does not require a measure of central tendency as a basis for calculation. To calculate the stability indicator, which always refers to a single business school, we compare each rank value with the remaining rank values of all relevant rankings. That means we compare a business school’s first rank value with its second rank value and take the difference as an absolute value. Since the maximum difference between rank values when ranking six institutions is 5, the rank difference value is always an integer between 0 and 5. We then compare the first rank value to the third rank value and take the difference. We iterate these comparisons up to the last rank value of the relevant rankings. Next, we take the second rank value and compare it to the remaining rank values (third rank value to last rank value) in the same way. We then take the next rank value and iterate in the same way until we have used all relevant rank values as a base value for comparison with the remaining rank values. Finally, we sum all the absolute difference values obtained and divide this sum by the number of comparisons made to get the relevant stability indicator. To address the differences between publication-based and citation-based rankings, we first calculate a publication-based and a citation-based stability indicator for each business school, which we then combine into an overall stability indicator. This indicator also has values between 0 and 5. The lower this value, the higher the stability of the rankings considered with regard to a business school.

To answer research question 4, i.e., to check the stability of ranking results in case of variations in ranking methods, we calculate rank correlation coefficients. In particular, we check the stability in the event of a change from publication-based to citation-based rankings, as well as in the event of a change in the data source, the use of full counting versus adjusted counting, and the consideration of all researchers versus only of the top researchers.

To answer research question 5, we use the results regarding research questions 2 to 4 to discuss whether it may be useful to conduct a university ranking solely on the basis of a single (overall) indicator. This discussion can be found in the *Conclusions* section and involves the established international university rankings, which often use such a single indicator.

## Results and discussion

When it comes to the visibility of business administration research at the selected Austrian universities in WoS and Scopus, Table 5 shows the number of researchers assigned to each business school, as well as how many of those researchers were found in WoS and Scopus (i.e., whether at least one publication could be found for a researcher). We were able to find at least one publication for 227 out of 283 researchers (80%) in WoS. The results are slightly better in Scopus (85%) which aligns with past studies that also showed a higher coverage for Scopus [95, 96]. In relative numbers, Vienna has the highest share of researchers with records found in WoS and Scopus (95% each) and thus the highest *business administration* research visibility out of the six Austrian universities. Innsbruck scores second place (85% in WoS and 88% in Scopus). Vienna is also the only business school which has identical numbers of found individuals for both WoS and Scopus. The university with the lowest visibility of business administration is Klagenfurt, preceded by Graz.

**Table 5:** Business school rankings based on visibility in WoS and scopus.

Business school	Number of researchers								
	total	with publications found							
		absolute no. (and rank)				in % of total (and rank)			
		WoS		Scopus		WoS		Scopus	
Graz	34	23	5	27	5	68%	5	79%	5
Innsbruck	41	35	3	36	3	85%	2	88%	2
Klagenfurt	20	13	6	15	6	65%	6	75%	6
Linz	48	36	2	41	2	75%	4	85%	3
Vienna	37	35	3	35	4	95%	1	95%	1
VUEB	103	85	1	86	1	83%	3	83%	4
**Total**:	**283**	**227**		**240**		**80%**		**85%**	

*Implications for research practice*: In terms of visibility in WoS and Scopus, the most important influencing factor for a good ranking of a business school is a high proportion of active researchers who–during the observation period–have published at least one paper that is recorded in WoS or Scopus. The prerequisite for this factor is usually the publishing of articles in English, since German-language journals are hardly included in the two relevant databases. The total number of publications from a business school (regardless of whether they are standardized by size or not) only plays a subordinate role in this ranking, since particularly active researchers cannot compensate for the poor research performance of inactive colleagues here.

*Stability*: It can be noted that the ranks of the six business schools remain largely the same when changing the data source from WoS to Scopus. This is probably mainly due to the relationship between the visibility of the associated researchers being similar in WoS and Scopus at all considered business schools. It would be different if there were many researchers at individual business schools who publish in journals that are, e.g., not included in WoS but are included in Scopus. In our case, the number of such researchers is between 0 (Vienna) and 5 (Linz). When switching from rankings based on size-dependent indicators (Table 5: absolute no.) to those based on size-independent indicators (Table 5: in % of total), there are indeed significant ranking differences. For example, the VUEB slips from first to third (WoS) and fourth place (Scopus), which we believe is justified since large institutions are unjustifiably preferred when using size-dependent indicators.

### Publication-based rankings

For the quantitative analysis of publication output, one of the indicators most commonly used is the *publication rate in a certain time period* [4]. For the time period 2008 to 2017 and when applying full (adjusted) counting (for calculation explanation, refer to the sub-section *S2 Calculation of adjusted publication count* in the S1 Appendix), we found a total of 1,937 (789) publications in WoS, and 2,530 (1,030) publications in Scopus (see Table 6). Therefore, analogous to the business schools’ higher visibility in Scopus, their research coverage is also higher in Scopus, with 593 (241) publications more found in Scopus than in WoS.

**Table 6:** Business school rankings based on number of publications.

Business School	Total number of publications								Total no. of res.	Number of publications per researcher							
	full counting				adjusted counting					full counting				adjusted counting			
	WoS		Scopus		WoS		Scopus			WoS		Scopus		WoS		Scopus	
	count	rank	count	rank	count	rank	count	rank		count	rank	count	rank	count	rank	count	rank
Graz	185	5	253	5	80	4	113	5	34	5.4	4	7.4	4	2.4	4	3.3	3
Innsbruck	282	3	385	3	117	3	155	3	41	6.9	3	9.4	2	2.8	2	3.8	2
Klagenfurt	81	6	126	6	36	6	55	6	20	4.1	5	6.3	5	1.8	5	2.8	5
Linz	187	4	290	4	71	5	116	4	48	3.9	6	6.0	6	1.5	6	2.4	6
Vienna	474	2	600	2	206	2	250	2	37	12.8	1	16.2	1	5.6	1	6.8	1
VUEB	728	1	876	1	279	1	340	1	103	7.1	2	8.5	3	2.7	3	3.3	4
**Sum**:	**1,937**		**2,530**		**789**		**1,030**		**Total**:	**6.8**		**8.9**		**2.8**		**3.6**	

In all rankings based on the *total number of publications*, the VUEB is clearly in first place, always followed by Vienna. If, however, the size (i.e., the number of researchers) is considered, Vienna takes first place in all relevant rankings, while the VUEB falls back to places 2, 3 (in two cases), and even 4. Linz ranks last in all four size-independent rankings, and Klagenfurt scores just slightly better. Since the consideration of the size of the compared units is widespread in research [17, 47, 109–111] and is to be preferred with regard to the reasonableness (fairness) of comparisons between comparison units of (considerably) different sizes, such as the six business schools under consideration, we carried out all subsequent rankings (including all citation-based rankings) solely on a scaled (size-independent) basis (for calculation explanation, refer to the sub-section *S3 Calculation of publication count per researcher* in the S1 Appendix).

### Implications for research practice

With regard to the four size-independent rankings based on the number of publications (publications per researcher), it should be noted that a business school performs better the more its researchers publish on average. However, the lack of research performance by non-active researchers can very well be compensated by an above-average research performance by active researchers.

### Stability

The ranks of the individual business schools are pretty stable in all rankings based on the publications per researcher. There are no changes at all for three business schools (Klagenfurt, Linz and Vienna), and for the remaining three business schools, there is merely a slight change by one rank. The results would be different if there were many researchers at individual institutions who, e.g., often publish in journals that are not included in WoS but in Scopus, or who publish particularly often with many co-authors.

Table 7 contains business school rankings by *number of pages per researcher* (for calculation explanation, refer to the sub-section *S4 Calculation of number of pages per researcher* in the S1 Appendix). Just as we found more publications in Scopus than in WoS (2,530 compared to 1,937; see Table 6), the page numbers per researcher based on Scopus are also higher than those based on WoS (165 compared to 128), even when the page numbers are adjusted (67 compared to 52). Identical to the rankings by the number of publications per researcher (see Table 6), Vienna scores at the top in all four rankings by number of pages per researcher, and Linz and Klagenfurt score last.

**Table 7:** Business school rankings based on number of pages per researcher.

Business school	Number of pages per researcher							
	full counting				adjusted counting			
	WoS		Scopus		WoS		Scopus	
	count	rank	count	rank	count	rank	count	rank
Graz	92	4	128	4	41	4	58	4
Innsbruck	128	3	174	2	53	2	70	2
Klagenfurt	86	5	114	6	40	5	49	5
Linz	78	6	117	5	30	6	46	6
Vienna	234	1	296	1	103	1	124	1
VUEB	133	2	159	3	50	3	63	3
**Total**:	**128**		**165**		**52**		**67**	

### Implications for research practice

When doing page-based rankings, a business school performs better the more (in terms of pages) its authors publish. However, a relatively small number of publications can hardly be compensated by a significantly above-average length of these publications, since there are often page limits (character limits) for individual journal articles.

### Stability

As can be seen in Table 7, Graz and Vienna have stable results across all four rankings, while the other business schools have largely stable ranking results, as well, with only one change in ranks.

We further ranked the six business schools by their number of fairly active researchers (defined as professors with five or more publications in WoS or Scopus) and very active researchers (defined as professors with ten or more publications in WoS or Scopus). Table 8 contains the according results. To put this into perspective, it should be noted that fairly (very) active here means that an average of at least 0.5 (one) article(s) per year were (was) published during the observation period. Here, Vienna again claims the top spot in all four rankings while Linz consistently scores at the bottom. A comparative look at Table 5 reveals that while there are many active researchers at Linz, Linz only has a few fairly active or very active researchers. The overall higher values in Scopus align with our previous results.

**Table 8:** Business school rankings based on the share of highly active researchers.

Business school	Relative number of researchers with five or more publications found				Relative number of researchers with ten or more publications found			
	WoS		Scopus		WoS		Scopus	
	count	rank	count	rank	count	rank	count	rank
Graz	44%	5	50%	5	24%	4	38%	2
Innsbruck	54%	2	61%	2	24%	3	34%	4
Klagenfurt	45%	4	55%	4	10%	6	35%	3
Linz	27%	6	35%	6	10%	5	19%	6
Vienna	73%	1	76%	1	46%	1	54%	1
VUEB	53%	3	58%	3	28%	2	32%	5
**Total**:	**50%**		**56%**		**25%**		**34%**	

### Implications for research practice

When it comes to the main factor influencing the rankings in Table 8, reference is made to the comments on Table 5; with the difference that less active researchers (i.e., those with at least one but less than five respective ten publications in the observation period) are not relevant here, but only fairly or very active researchers (i.e., those with at least five or ten publications).

### Stability

It can be seen that there are significant fluctuations between the four rankings at individual business schools. For example, Graz performs much better if the focus is not on the fairly active but on the very active researchers. For Innsbruck, the situation is exactly the opposite.

Our final publication-based rankings are based on the aggregated publication output of the business schools’ top five researchers (refer to Table 9; in our data set, the same researchers make up the top five by full counting in both databases). Our rankings confirm the dominance of Vienna and the poor position of Klagenfurt.

**Table 9:** Business school rankings based on the top five most publishing researchers’ publication counts.

Business school	Aggregated number of publications (top five researchers)							
	full counting				adjusted counting			
	WoS		Scopus		WoS		Scopus	
	count	rank	count	rank	count	rank	count	rank
Graz	87	5	106	5	40	4	48	4
Innsbruck	121	3	153	3	44	3	54	3
Klagenfurt	45	6	68	6	19	6	29	6
Linz	88	4	118	4	31	5	46	5
Vienna	200	1	244	1	90	1	101	1
VUEB	170	2	192	2	56	2	67	2

### Implications for research practice

In all four rankings based on the (publications of the) top five researchers, a business school performs better the more these five researchers stand out. It should be noted that this ranking omits the consideration of the research performance of the business schools’ other researchers. For this ranking, it is therefore irrelevant whether the remaining researchers are also fairly or even very active researchers or completely inactive researchers. It should also be noted that the pool of researchers at smaller institutions is inherently smaller, reducing the possibility of having high-performing researchers.

### Stability

The positions of the six business schools are highly stable in the four rankings in Table 9. There is only a shift of one rank between two business schools (Graz and Linz) when adjusted counting is applied instead of full counting. The ranks, however, would not be as stable if there were top five researchers at individual business schools who often publish in journals that are not included in WoS but in Scopus, or who publish particularly often with many co-authors.

### Citation-based rankings

From WoS, we retrieved a total of 38,827 citations (including self-citations) for 227 found researchers and their 1,937 found publications, which is 20 citations per publication and 137 citations per researcher (see Table 10). Excluding self-citations, the total citation count decreases slightly to 37,415, which means that only 3.6% (i.e., 1,412 citations) of all citations retrieved from WoS are self-citations. From Scopus we retrieved a total of 58,301 citations (including self-citations) for 240 found researchers and their 2,530 publications, which is 23 citations per publication and 206 citations per researcher (see Table 10). Excluding self-citations, the total citation count drops to 51,195, which means that 12.2% (i.e., 7,106 citations) of all citations retrieved from Scopus are self-citations. The higher value is due to the fact that Scopus also considers citations from co-authors as self-citations. The number of retrieved citations is significantly higher in Scopus, with almost 20,000 more citations found than in WoS. This aligns with Scopus’ higher publication counts and database visibility shown so far. Since we could hardly determine any ranking result differences when including and excluding self-citations, the following citation-based analyses are solely based on citation counts with self-citations included.

**Table 10:** Business school rankings based on average citation count.

Business school	Average citation count															
	citations per researcher								citations per publication							
	full counting				adjusted counting				full counting				adjusted counting			
	WoS		Scopus		WoS		Scopus		WoS		Scopus		WoS		Scopus	
	count	rank	count	rank	count	rank	count	rank	count	rank	count	rank	count	rank	count	rank
Graz	54	5	78	6	23	4	33	6	10	6	11	6	4	6	4	6
Innsbruck	148	3	214	3	61	3	84	3	22	2	23	2	9	2	9	2
Klagenfurt	50	6	98	5	23	5	42	4	12	5	16	5	6	4	7	4
Linz	64	4	114	4	19	6	36	5	16	4	19	4	5	5	6	5
Vienna	237	1	353	1	92	1	128	1	19	3	22	3	7	3	8	3
VUEB	175	2	256	2	64	2	92	2	25	1	30	1	9	1	11	1
**Total**:	**137**		**206**		**52**		**75**		**20**		**23**		**8**		**8**	

**Fig 2:**
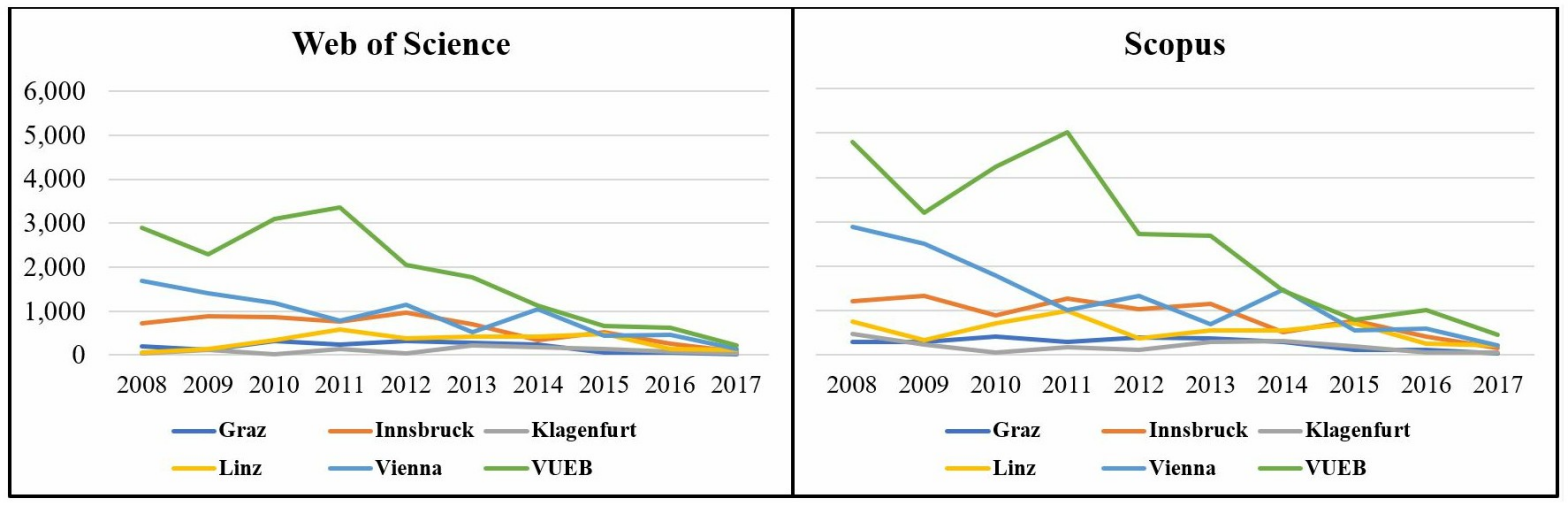
Citations per business school and publication year.

Fig 2 shows the distribution of the citations to the publications from the various publication years by business school in WoS and Scopus. For almost all business schools, there is a slight upward trend for citations to publications from 2009 to 2011, with the VUEB dominating in both databases and showing a peak in citation counts for 2011-publications. For publications from 2014 onwards, however, its citation counts start to settle close to those of the other business schools. Vienna has its peak value for citations at the beginning of the observation period; after that, there is a sharp downward trend.

To solely have size-independent citation count rankings, we only included citation-based rankings according to average values. Table 10 contains the average (in relation to the number of researchers or publications) *full* and–for a fairer attribution of co-authorships–*adjusted* citation counts by business school (for calculation explanation, refer to the sub-sections *S5 Calculation of full citation count per researcher* and *S6 Calculation of adjusted citation count per researcher* in the S1 Appendix). Surprisingly, while Vienna leads all average citation rankings *per researcher*, it only occupies third place in the average citation rankings *per publication* (for calculation explanation, refer to the sub-section *S7 Calculation of full and adjusted citation count per publication* in the S1 Appendix). The reason for this is that although it accumulates many citations (regardless of whether they refer to many or a few publications), it also accumulates many articles–which is overall positive. In this respect, the relevance of the rankings based on the citations per publication must be put into perspective. Graz also performs (slightly) better in the rankings based on the citations per researcher, but still only occupies low ranks across all rankings. Innsbruck and the VUEB perform better by average citations per publication (where they are top ranked) than by averages per researcher (where they are also ranked above average), while Klagenfurt and Linz are largely equally (poorly) placed in both rankings.

### Implications for research practice

The values contained in Table 10 are average values. For these, it is irrelevant whether numerous authors or publications assigned to a business school are cited weakly to moderately, or whether only a few individual authors or publications are cited highly to very highly. In extreme cases, a business school can achieve a top position in these rankings via a single extremely heavily cited publication by a single author. Just as in our publication-based analyses, Scopus has higher values than WoS in our citation-based analyses, too.

### Stability

The ranks of the six business schools remain relatively unchanged when moving from WoS to Scopus. There are larger changes when switching from full to adjusted counting, and even larger–as was expected based on previous results–when switching from citations per researcher to citations per publication.

In Table 11, we rank each business school using the aggregated citation counts of their top five assigned researchers by citation counts. The results underline once more the dominance of the VUEB and Vienna, which achieve four to nine times higher scores than Klagenfurt and Graz.

**Table 11:** Business school rankings based on the top five most cited researchers’ citation counts.

Business school	Aggregated number of citations (top five most cited researchers)							
	full counting				adjusted counting			
	WoS		Scopus		WoS		Scopus	
	count	rank	count	rank	count	rank	count	rank
Graz	1,008	5	1,289	6	421	5	561	6
Innsbruck	3,574	3	4,833	3	1,452	3	1,895	3
Klagenfurt	753	6	1,604	5	331	6	674	5
Linz	2,106	4	3,768	4	602	4	1,123	4
Vienna	5,835	2	8,731	2	2,067	2	2,868	2
VUEB	6,798	1	9,857	1	2,293	1	3,255	1

### Implications for research practice

In all four rankings based on the citations of the top five most cited researchers, a business school performs better the more these five researchers stand out with regard to citations. For further details, refer to our corresponding comments on Table 9.

### Stability

Just as in the publication-based rankings based on the top five most publishing researchers (see Table 9), the results here are also highly stable. Again, there is only one shift, but this time, when changing from WoS to Scopus and with regard to Graz and Klagenfurt.

Table 12 contains the rounded off *h*-indices as an *average per researcher*, as well as *per business school*. (For calculation explanation, refer to the sub-section *S8 Calculation of h-indices* in the S1 Appendix). In our two rankings by average *h*-indices *per researcher*, Vienna is at the top, followed by the VUEB. In the two rankings based on *h*-indices *per business school*, the VUEB is in first place, followed by Vienna in WoS and Innsbruck in Scopus. Graz and Klagenfurt, on the contrary, consistently occupy the bottom ranks.

**Table 12:** Business school rankings based on *h*-indices.

Business school	*h*-index							
	average per researcher				per business school (formula)			
	WoS		Scopus		WoS		Scopus	
	count	rank	count	rank	count	rank	count	rank
Graz	2	4	3	5	16	5	19	5
Innsbruck	3	3	5	3	25	3	30	2
Klagenfurt	2	5	2	6	12	6	12	6
Linz	2	6	3	4	20	4	23	4
Vienna	5	1	7	1	26	2	29	3
VUEB	4	2	5	2	51	1	59	1

### Implications for research practice

When ranking by *h*-indices, it should be noted that individual researchers who have many heavily cited articles and who also have very high *h*-indices can have some influence on the results. However, this influence is not as strong as when ranking by average citation counts. Conversely, individual heavily cited articles by one or more researchers can only have a limited (positive) influence on the results, since those alone do not lead to high *h*-indices.

*Stability*: While the ranking orders vary between our two *h*-index calculations, those between WoS and Scopus are identical or very similar, since the *h*-index in particular is not influenced by outliers [112]. One noticeable difference is that for both calculation methods, Innsbruck scores better in Scopus.

### Stability of rankings

In the sub-sections *Publication-based rankings* and *Citation-based rankings*, we discussed the stability of the results within the individual tables. In the following, we use all of our ranking results to show the overall stability of the results. Tables 13 and 14 list the ranking values of our conducted publication- and citation-based analyses. From Table 5, we considered solely the ranking results based on the proportion of researchers with publications found in % of the total–these ranks represent T5-1 (in % of total in WoS) and T5-2 (in % of total in Scopus) in Table 13. (The other two rankings that Table 5 contains were for demonstration purposes only.) From Tables 6 to 9, we included all ranking results in Table 13. Table 14 includes all ranking results from Tables 10 to 12.

**Table 13:** Stability of rankings (publication-based rankings).

University	Publication-based rankings																					
	Table 5		Table 6								Table 7				Table 8				Table 9			
	a)		*b)*				c)				*d)*				e)		f)		g)			
			fc		ac		fc		ac		fc		ac						fc		ac	
	W	S	W	S	W	S	W	S	W	S	W	S	W	S	W	S	W	S	W	S	W	S
	1	2	1	2	3	4	5	6	7	8	1	2	3	4	1	2	3	4	1	2	3	4
Graz	**5**	**5**	*5*	*5*	*4*	*5*	**4**	**4**	**4**	**3**	*4*	*4*	*4*	*4*	**5**	**5**	**4**	**2**	**5**	**5**	**4**	**4**
Innsbruck	**2**	**2**	*3*	*3*	*3*	*3*	**3**	**2**	**2**	**2**	*3*	*2*	*2*	*2*	**2**	**2**	**3**	**4**	**3**	**3**	**3**	**3**
Klagenfurt	**6**	**6**	*6*	*6*	*6*	*6*	**5**	**5**	**5**	**5**	*5*	*6*	*5*	*5*	**4**	**4**	**6**	**3**	**6**	**6**	**6**	**6**
Linz	**4**	**3**	*4*	*4*	*5*	*4*	**6**	**6**	**6**	**6**	*6*	*5*	*6*	*6*	**6**	**6**	**5**	**6**	**4**	**4**	**5**	**5**
Vienna	**1**	**1**	*2*	*2*	*2*	*2*	**1**	**1**	**1**	**1**	*1*	*1*	*1*	*1*	**1**	**1**	**1**	**1**	**1**	**1**	**1**	**1**
VUEB	**3**	**4**	*1*	*1*	*1*	*1*	**2**	**3**	**3**	**4**	*2*	*3*	*3*	*3*	**3**	**3**	**2**	**5**	**2**	**2**	**2**	**2**

Values not in bold but in italics = Results of rankings that we would not recommend for performance comparisons.

W = Web of Science (WoS)

S = Scopus

fc = full counting

ac = adjusted counting

a) = Proportion of researchers with publications found (in % of total)

b) = Total number of publications

c) = Number of publications per researcher

d) = Number of pages per researcher

e) = Relative number of researchers with five or more publications found

f) = Relative number of researchers with ten or more publications found

g) = Aggregated number of publications (top five researchers)

**Table 14:** Stability of rankings (citation-based rankings).

University	Citation-based rankings															
	Table 10								Table 11				Table 12			
	a)				*b)*				c)				*d)*		*e)*	
	fc		ac		fc		ac		fc		ac		W	S	W	S
	W	S	W	S	W	S	W	S	W	S	W	S				
	1	2	3	4	5	6	7	8	1	2	3	4	1	2	3	4
Graz	**5**	**6**	**4**	**6**	*6*	*6*	*6*	*6*	**5**	**6**	**5**	**6**	*4*	*5*	*5*	*5*
Innsbruck	**3**	**3**	**3**	**3**	*2*	*2*	*2*	*2*	**3**	**3**	**3**	**3**	*3*	*3*	*3*	*2*
Klagenfurt	**6**	**5**	**5**	**4**	*5*	*5*	*4*	*4*	**6**	**5**	**6**	**5**	*5*	*6*	*6*	*6*
Linz	**4**	**4**	**6**	**5**	*4*	*4*	*5*	*5*	**4**	**4**	**4**	**4**	*6*	*4*	*4*	*4*
Vienna	**1**	**1**	**1**	**1**	*3*	*3*	*3*	*3*	**2**	**2**	**2**	**2**	*1*	*1*	*2*	*3*
VUEB	**2**	**2**	**2**	**2**	*1*	*1*	*1*	*1*	**1**	**1**	**1**	**1**	*2*	*2*	*1*	*1*

Values not in bold but in italics = Results of rankings that we would not recommend for performance comparisons.

W = Web of Science (WoS)

S = Scopus

fc = full counting

ac = adjusted counting

a) = Average citation count–citations per researcher

b) = Average citation count–citations per publication

c) = Aggregated number of citations (top five most cited researchers)

d) = h-index–average per researcher

e) = h-index–average per business school (formula)

Table 13 shows that Vienna is in first place in all rankings that we consider useful for a performance comparison in practice (those that we did not consider in our calculations are marked in italics and not bold)–thus in a total 14 rankings results. The ranks of the other five business schools are not quite as stable. Using mode as a measure of central tendency, Innsbruck and the VUEB would tie for second place. Innsbruck occupies this position in seven of the 14 relevant cases, the VUEB in six. Innsbruck performs particularly well in rankings based on publications per researcher, while the VUEB performs particularly well in those based on publications by the top five most publishing researchers. The other three business schools are in the lower ranks, with Graz doing slightly better than Klagenfurt and Linz. Graz performs best at rankings based on publications per researcher, Klagenfurt at rankings based on highly active researchers, and Linz at rankings based on visibility.

Table 14 reveals results (again ignoring the values marked in italics but not bold) that are similar to those in Table 13. Vienna and the VUEB share the top position here, however, while Innsbruck is in third place. Out of the other three universities, Linz fares best this time, and Graz the worst.

To answer research question 3, we calculated a publication-based (PA) stability indicator, a citation-based (CA) stability indicator, and an overall stability indicator for each business school (see Table 15). (For calculation explanation, refer to the sub-section *S9 Calculation of the three stability indicators* in the S1 Appendix). For these calculations, however, we only used those variants that we consider useful for a performance comparison in practice (thus all values marked bold in Tables 13 and 14). The results for the total stability index show that the rankings of the individual business schools are relatively stable. The total stability values range between 0.31 (Vienna) and 1.13 (VUEB). For Vienna, for example, this means that there is an average fluctuation of 0.31 (ranks) in the 22 relevant rankings. Given that our stability value range is between 0 and 5, this result can be interpreted as a very high stability. A look at the two partial stability indices (i.e., PA and CA) shows that in both cases, there is a business school that achieves maximum stability, i.e., a value of 0 (which is Vienna with regard to the publication analyses, and Innsbruck with regard to the citation analyses).

**Table 15:** Stability values (publication analysis (PA), citation analysis (CA), and total).

Business school	Stability PA		Stability CA		Stability total	
	sum of obtained difference values	stability value	sum of obtained difference values	stability value	sum of obtained difference values	stability value
Graz	85	0.93	23	0.82	250	1.08
Innsbruck	62	0.68	0	0	126	0.55
Klagenfurt	95	1.04	22	0.79	213	0.92
Linz	102	1.12	19	0.68	253	1.10
Vienna	0	0	16	0.57	72	0.31
VUEB	94	1.03	16	0.57	262	1.13

To answer research question 4, i.e., to check the stability of ranking results in case of variations in ranking methods, we calculated rank correlation coefficients. Table 16 shows that switching from publication-based to citation-based rankings has the greatest impact on ranks. If rankings based on the citations per researcher are used instead of rankings based on the publications per researcher, Vienna remains the undisputed number one, but overall, VUEB and Innsbruck swap positions. Graz performs significantly worse after this change, Linz significantly better. If the citations of the top five most cited researchers are considered instead of the publications of the top five most publishing researchers, Vienna loses its top position to VUEB. After this change, Graz does noticeably worse yet again. Switching from a ranking based on all considered researchers to one based only on the top five researchers also has a significant impact on the ranking results of the individual business schools. In our case study, the VUEB and Linz would benefit from such a change, while Klagenfurt would clearly do worse. The used data source as well as the treatment of co-authorships, in contrast, only have a minor influence on the ranking results. A change from WoS to Scopus would most likely have (negative) effects on VUEB, while a change from full to adjusted counting would most likely have (positive) effects on Graz.

**Table 16:** Stability of ranking results in case of ranking method variations.

Analysis type	Rank correlation coefficient (Spearman)			
	PA rankings vs. CA rankings	WoS vs. Scopus	Full counting vs. adjusted counting	All researchers vs. top five researchers
**PA** (Publication analysis)	-	0.91	0.94	0.81
**CA** (Citation analysis)	-	0.93	0.93	0.87
**Total**:	0.81	0.91	0.93	0.84

## Conclusions and limitations

This article presents different ways in which business schools can be ranked and shows which of these variants make sense from our point of view. As part of our case study, we looked at (all) six business schools at public universities in Austria based on the research performance of 283 assigned professors. The study focused less on the specific ranking results and more on the different possibilities for such a ranking, as well as on methods for checking the stability of rankings. With regard to the possible procedures, we compared publication-based rankings with citation-based rankings that were as comparable as possible (e.g., we compared rankings based on publications per researcher with rankings based on citations per researcher).

We used WoS and Scopus as data sources for determining the publication and citation counts. Our review of the visibility of the considered researchers has shown that by no means all of the considered researchers have at least one publication in one or both of these databases over our set 10-year observation period. We found some differences in the universities’ *visibility* in the two databases. 80% of researchers have at least one publication found in WoS, and 85% in Scopus–the higher visibilities of Scopus aligns with past studies [94–96]. In WoS, 50% of the professors have five or more publications retrieved, while it is 56% in Scopus. We also found a higher number of publications with German as publication language from Scopus than from WoS.

In total, we conducted 22 publication-based and 16 citation-based rankings. Out of these, we considered 14 publication-based and 8 citation-based rankings (thus a total of 22 rankings) useful for performance comparison between different business schools. The remaining 16 rankings appear to be informative but less suitable for such a comparison. For example, size-dependent rankings (in our case based on the total number of publications) favour larger institutions, while rankings based on page numbers prioritize quantity over quality, and rankings based on citations per publication can lead to distortions if there are overall only a few publications. In addition to the sheer number of publications and citations, important factors influencing the (useful) rankings were the distribution of publications among the relevant professors (in terms of visibility and highly active researchers), and the average number of co-authors (in terms of adjusted counting).

On the one hand, we checked the stability of the rankings with regard to the individual business schools using tabular comparisons, which already show that the ranks of the individual business schools are relatively stable. However, such tabular comparisons only make sense in the case of a limited number of comparison objects, since they otherwise quickly become confusing. On the other hand, stability indicators appear to be more suitable for a larger number of comparison objects. For the present study, we have developed our own stability indicator, which has proven itself in practical use. The corresponding stability values for the individual business schools also indicate high stability.

To check how stable the ranking results are in case of variations in ranking methods, we calculated rank correlation coefficients. These reveal that a change from publication-based to comparable citation-based rankings has the greatest impact. Also of note is a shift from rankings based on all relevant researchers to rankings based only on the top researchers. However, the results remain relatively stable if the data source (Scopus instead of WoS) or the way in which co-authorships are taken into account (adjusted instead of full counting) is changed. Our presented publication and citation analyses reveal some differences between WoS and Scopus when ranking the six selected business schools–among other factors due to higher visibilities in Scopus. The overall business school ranks, however, are similar in both databases. Those business schools with higher ranks in WoS usually also achieve high ranks in Scopus.

As a summary of our empirical study and with regard to research question 5, we explicitly recommend using several ranking variants for institutional rankings in the university sector, which can then be compared with each other using stability indicators, such as the ones we developed. If there is sufficient stability, there is the possibility of reducing the number of variants again (e.g., with regard to publications). In contrast to many existing ranking systems, which focus on one variant (indicator), there will usually still be several variants with different results. Even in the case of our small sample of just six universities, the ranking results were not sufficiently stable to be able to reduce the ranking to one variant. The established Leiden rankings already follow a similar multiple approach, but without attempting to reduce variants with similar results. The Leiden rankings are also limited to citation analysis.

Therefore, the main contribution of our approach to existing international university rankings is that it demonstrates that no composite indicators should be used. Instead, separate and more comprehensive rankings for each considered indicator should be performed. A composite indicator is not only problematic for methodological reasons, it also often ‘hides’ single weak-points of the evaluation objects.

Our ranking results for the six Austrian universities correspond–if available–to those of the 2022 ARWU *Global Ranking of Academic Subjects* in *Business Administration* [113], the 2023 QS in *Business and Management Studies* [114], the 2023 SIR in *Business*, *Accounting and Management* [115], the 2023 THE in *business and economics* [116], the 2021–2022 UMR *by Subject* in *Business studies* [117], and the 2022–2023 U.S. NWR in *Economics and Business* [118].

Research performance in existing business school rankings is often limited to publications in (business) management journals (see [32] and [30]). Even if the journal lists used are very extensive, publications by researchers working at business schools are by no means limited to this research area. Our study relied on individual researchers to determine research performance, which avoids this problem.

With regard to accreditation, it should be noted that of the six business schools considered, only two were accredited by one of the organizations mentioned in the introduction: Klagenfurt by the AACSB and VUEB by AACSB, AMBA and EFMB, giving it the triple crown status. While VUEB does well in our rankings, Klagenfurt is usually in last place. In this respect, our study did not reveal any connection between accreditation and above-average research performance.

Finally, we would like to point out some limitations of our study. The main limitation is the specific and relatively small population–only six business schools from Austria. A restriction to such a small number of subjects of investigation is resource-saving and justifiable with regard to our main research goal–i.e., to carry out a ranking using not just one method, but to analyse the resulting effects when using different methods. If one compares our approach and results with those of the studies on the ranking of business schools listed in the introduction, Kumar and Kundu [27] evaluated 600 institutions, but only on the basis of 900 articles, as their study was limited to publications in only three journals. In contrast, we used all publications (contained in the databases used) for an equally long study period, resulting in a number of 1,937 (WoS) and 2,530 (Scopus) publications for only six institutions. It would be optimal to combine the data sources we used with the variety of institutions used by Kumar and Kundu [27], although this would be very resource intensive.

With regard to the data retrieval in the two databases used, we cannot completely rule out the possibility that technical limitations may have affected the accuracy of our bibliometric study by incorrectly attributing publications and citations. At least misclassification, which can occur when researching at business school level, was avoided because we conducted research at the level of individual researchers.

Even at discipline level, such as business administration (management), a further sub-division could make sense. Since business administration is strongly interdisciplinary, the sub-disciplines usually have different publication and citation behaviors. In our case, also the publication language had some impact. For instance, financial accounting departments published more often in German [119]. Accordingly, business schools with big accounting departments were discriminated to those in which this sub-discipline was only weaker positioned.

With regard to the ranking variants used, it should be noted that we did not include impact factors (JIFs) in our ranking analyses. Using JIFs as measures for evaluating individuals has been labelled a misuse by Garfield [107] who argues that looking up their articles’ citation counts is a better assessment option on individual level, since even within a single journal, there is a wide variation from article to article, and the contribution of articles to a journal’s impact is uneven [120]. Avoiding the use of journal-based metrics (e.g., JIFs) to measure of the quality of individual research articles has also been recommended by the San Francisco Declaration on Research Assessment (DORA) [121] which consists of a set of recommendations for the assessment of research. The DORA stresses on focusing on article-level metrics, instead, to ease an assessment based on an article’s scientific content, rather than on its journal’s publication metrics [121]. Early literature and even journal editors have already shed a critical light on the usage of impact factors to assess scientific literature [120, 122, 123].

And finally, we should also point out a limitation with regard to a specific ranking variant, which we anyway presented exclusively for demonstration purposes without recommending it for other rankings: When using the number of pages for a ranking, there can be severe distortions due to different formats, font sizes and layouts. Therefore, it would be better to count the size in words or characters, but such data is rarely available. In general, using the number of pages as a performance indicator is viewed critically, because there is no connection between the length and quality of a paper. Highly respected journals, such as *Science* or *Nature*, mainly publish short articles [108]. For a citation-based ranking, the question of the general usefulness of a ranking based on the number of pages does not arise since there is not even a meaningful indicator for such a ranking.

## Supporting information

S1 Appendix (DOCX)
