# Supplementary material for: Possibilities for ranking business schools and considerations concerning the stability of such rankings
Source: PLoS One. 2024 Feb 15;19(2):e0295334. doi: 10.1371/journal.pone.0295334 (PMC10868868; doi:10.1371/journal.pone.0295334)
Supplement: S1 Appendix — (DOCX) [file pone.0295334.s001.docx]

# **Supporting information**

## **S1 Scopus field codes**

In Scopus, we used the *Author Name* field code mainly when there was no possible variation of the full name, e.g., Kirchler Michael, since it usually leads to more compact results than the broader *Author (AUTH)* field code. This quickens the initial search for the author-ID. Entering, e.g., “*AUTH ( jr )*” will return publications with “jr” in the last name, but also in the *first initial* fields, e.g., “Finn Jr., C.E.” and “Jenkins, J.R.”.

## **S2 Calculation of *adjusted* publication count**

We calculated each university’s *adjusted* publication count by summing up the adjusted publication counts of its assigned researchers. We adjusted each researchers’ publication count by each publication’s number of co-authors, using the common formula 1/n (n = number of co-authors) [21,124].

## **S3 Calculation of publication count *per researcher***

We calculated each business school’s publication count *per researcher* by dividing its total publication count by its total number of assigned researchers, including researchers who have zero publications retrieved from WoS or Scopus.

## **S4 Calculation of number of pages *per researcher***

We calculated each business school’s number of pages *per researcher* by dividing its *total* page number count by its total number of assigned researchers, including researchers who have zero publications retrieved from WoS or Scopus. The *adjusted* page number count of a business school is the sum of the *adjusted* page number counts of its assigned researchers. Each researcher’s *adjusted* page number count was calculated by dividing each of their publication’s page number count by the publication’s co-author count (including the researcher) and summing these results up for all publications retrieved for that researcher. We then accordingly calculated each business school’s *adjusted* *number of pages per researcher* by dividing its *adjusted* page number count by its total number of assigned researchers, including researchers who have zero publications retrieved from WoS or Scopus.

## **S5 Calculation of *full* citation count *per researcher***

We calculated the *full* citation count *per researcher* per business school by summing up the *full* citation counts (including self-citations) of all researchers assigned to the business school and dividing this sum by the number of researchers, including researchers who have zero publications or citations retrieved from WoS or Scopus (see column “Number of researchers - total” in Table 5). The average *total* *full* citation count (see bottom row in Table 10) for WoS (Scopus) is accordingly the sum of all *full* citations retrieved from WoS (Scopus) for all 283 researchers divided by 283.

## **S6 Calculation of *adjusted* citation count *per researcher***

Our calculations for the *adjusted* citation count *per researcher* per business school are based on the calculations by Gupta et al. (1998, p. 125) and Diodato (1994, p. 2f.). A researcher’s *adjusted* citation count is the sum of all of their publications’ adjusted citation counts. Each publication’s adjusted citation count is obtained by dividing its retrieved citation count by its number of co-authors (including the analysed researcher). To therefore obtain the *adjusted* citation count *per researcher* per business school, we summed up the *adjusted* citation counts of all researchers assigned to the business school and divided this sum by the number of researchers. The average *total* *adjusted* citation count for WoS (Scopus) is accordingly the sum of all *adjusted* citations retrieved from WoS (Scopus) for all 283 researchers divided by 283.

## **S7 Calculation of *full* and *adjusted* citation count *per publication***

We calculated the *full* citation count *per publication* per business school by summing up the *full* citation counts of all researchers assigned to the business school and dividing this result by the business school’s *full* publication count (see Table 6). We calculated the *adjusted* citation count *per publication* per business school by summing up the *adjusted* citation counts of all researchers assigned to the business school and dividing this result by the business school’s *full* publication count (see Table 6).

## **S8 Calculation of *h*-indices**

We calculated each business school’s average *h*-index *per researcher* by taking the sum of the *h*-indices of all researchers assigned to that business school and dividing this sum by the total number of those researchers, including researchers who have zero publications or citations retrieved from WoS or Scopus. We calculated each business school’s *h*-index via the *h*-index formula using calculations demonstrated by Khakbiz (2022).

## **S9 Calculation of the three stability indicators**

For the calculation of the three stability indicators, we compared each rank value with the remaining rank values. E.g., for the PA stability indicator of the VUEB, we compared its first rank value (Table 13, T5-1 = 3) with its second rank value (T5-2 = 4) and took the difference in absolute value (difference between T5-1 and T5-2 = 1). We then compared its first rank value (T5-1 = 3) with its third (relevant) rank value (T6-5 = 2) and took the difference (difference between T5-1 and T6-5 = 1). We then yet again compared its first rank value (T5-1 = 3) with its fourth rank value (T6-6 = 3) and took the difference (difference between T5-1 and T6-6 = 0). We iterated such comparisons until the last rank value of our PA (i.e., T9-4), and summed up the difference values obtained via those iterated comparisons with T5-1. Next, we took the second rank value (T5-2 = 4) and, in the same manner, compared it to the rank values to its right to eventually sum up all obtained differences (i.e., the difference between T5-2 = 4 and T6-5 = 2 is 2, the difference between T5-2 = 4 and T6-6 = 3 is 1, and so on). We then took the next rank value (i.e., T6-5 = 2) and iterated calculations in the same manner until we had used all of the VUEB’s rank values that we consider relevant (i.e., those written in bold in Table 13 and Table 14) as base value for comparison with values to its right. Lastly, we summed up all obtained difference values and divided this sum by the number of conducted comparisons to obtain the PA stability index for the VUEB. Accordingly, we obtained the PA stability indices for the other business schools as well as all CA stability indices in the same manner. Since each business school has 14 relevant PA rank values, the total number of conducted comparisons for the PA stability indices – and thus the denominator for our PA index calculations – is always 91, while that for the CA stability indices (with 8 relevant rank values) – and thus the denominator for our CA index calculations – is always 28. To obtain the *total* stability indices, we lined up and compared the PA and CA rank values with each other, and thus extended our comparison pool to 22 rank values per business school, and the number of conducted comparisons for each business school to 231. Therefore, e.g., the CA stability index for Klagenfurt is 22/28 = 0.79, and the total stability index for Vienna is 72/231 = 0.31 (see Table 15).
